# ROICellTrack: a deep learning framework for integrating cellular imaging modalities in subcellular spatial transcriptomic profiling of tumor tissues

**DOI:** 10.1093/bioinformatics/btaf152

**Published:** 2025-04-08

**Authors:** Xiaofei Song, Xiaoqing Yu, Carlos M Moran-Segura, Hongzhi Xu, Tingyi Li, Joshua T Davis, Aram Vosoughi, G Daniel Grass, Roger Li, Xuefeng Wang

**Affiliations:** Department of Biostatistics and Bioinformatics, H. Lee Moffitt Cancer Center & Research Institute, Tampa, FL 33612, United States; Department of Biostatistics and Bioinformatics, H. Lee Moffitt Cancer Center & Research Institute, Tampa, FL 33612, United States; Department of Pathology, H. Lee Moffitt Cancer Center & Research Institute, Tampa, FL 33612, United States; Department of Pathology, H. Lee Moffitt Cancer Center & Research Institute, Tampa, FL 33612, United States; Department of Biostatistics and Bioinformatics, H. Lee Moffitt Cancer Center & Research Institute, Tampa, FL 33612, United States; Department of Biostatistics and Bioinformatics, H. Lee Moffitt Cancer Center & Research Institute, Tampa, FL 33612, United States; Department of Pathology, H. Lee Moffitt Cancer Center & Research Institute, Tampa, FL 33612, United States; Department of Radiation Oncology, H. Lee Moffitt Cancer Center & Research Institute, Tampa, FL 33612, United States; Department of Genitourinary Oncology, Moffitt Cancer Center, Tampa, FL 33612, United States; Department of Biostatistics and Bioinformatics, H. Lee Moffitt Cancer Center & Research Institute, Tampa, FL 33612, United States

## Abstract

**Motivation:**

Spatial transcriptomic (ST) technologies, such as GeoMx Digital Spatial Profiler, are increasingly utilized to investigate the role of diverse tumor microenvironment components, particularly in relation to cancer progression, treatment response, and therapeutic resistance. However, in many ST studies, the spatial information obtained from immunofluorescence imaging is primarily used for identifying regions of interest (ROIs) rather than as an integral part of downstream transcriptomic data analysis and interpretation.

**Results:**

We developed ROICellTrack, a deep learning-based framework that better integrates cellular imaging with spatial transcriptomic profiling. By analyzing 56 ROIs from urothelial carcinoma of the bladder and upper tract urothelial carcinoma, ROICellTrack identified distinct cancer–immune cell mixtures, characterized by specific transcriptomic and morphological signatures and receptor–ligand interactions linked to tumor content and immune infiltrations. Our findings demonstrate the value of integrating imaging with transcriptomics to analyze spatial omics data, improving our understanding of tumor heterogeneity and its relevance to personalized and targeted therapies.

**Availability and implementation:**

ROICellTrack is publicly available at https://github.com/wanglab1/ROICellTrack.

## 1 Introduction

Recent advances in spatial transcriptomics (STs) have expanded our ability to investigate the molecular architecture of normal and tumor tissues in their native microenvironment. Unlike bulk or single-cell RNA-sequencing approaches, ST technologies preserve spatial context, enabling researchers to analyze gene expression patterns in specific compartments or cellular niches. Among these technologies, the GeoMx Digital Spatial Profiler (DSP) by NanoString has seen increased use in the cancer genomics community for its ability to enable high-resolution transcriptomic profiling of histologically defined regions of interest (ROIs) (Van and Blank 2019). This approach bridges the gap between traditional histology and next-generation sequencing, allowing for precise molecular characterization of spatially distinct tumor microenvironments. GeoMx DSP is particularly advantageous for formalin-fixed, paraffin-embedded (FFPE) samples, which are widely used in cancer clinical research but pose greater challenges for traditional RNA-sequencing. Within this platform, H&E and multiplex immunofluorescence (mIF) staining guide the selection of ROIs, often determined by a pathologist, for targeted transcriptome assays, which are then analyzed using next-generation sequencing (NGS) readouts. However, a key limitation of most current applications of GeoMx DSP is the underutilization of cellular imaging data beyond ROI selection. While mIF imaging plays a crucial role in defining ROIs, its potential for integrating morphological and transcriptomic features in downstream bioinformatics analysis remains largely unexplored.

A key task in analyzing ST data for solid tumors is capturing the complexity of the tumor microenvironment (TME), which consists of diverse populations of cancer, immune, and their adjacent stromal cells. For example, in GeoMx DSP, pan-cytokeratin (PanCK), a biomarker for epithelial cells, is commonly used to differentiate tumor cells (PanCK^+^) from stromal components (PanCK^−^). Understanding the cellular composition and spatial organization of these components is essential for understanding mechanisms of tumor progression, immune evasion, and therapeutic resistance. The current bioinformatics downstream analysis pipeline of GeoMx data primarily relies on transcriptomic deconvolution methods to estimate cellular composition (Danaher *et al.* 2022), yet these approaches have limitations in accurately estimating cellular compositions across tissue types (Li *et al.* 2023, Garmire *et al.* 2024), an intrinsic limitation of gene expression reference-based approach, particularly for underrepresented cell populations and cells with transitional states. Furthermore, the spatial proximity and interactions between different cell types, which influence cellular communication and gene expression patterns, are missing from the deconvolution analysis. Essentially, these so-called mini-bulk analyses treat spatially defined ROIs similarly to bulk tumor analyses, failing to fully capture the spatial context and heterogeneity of the tumor microenvironment.

To address these limitations, we developed ROICellTrack, a bioinformatics pipeline that integrates *in situ* imaging with transcriptomics to enhance spatial molecular profiling and improve the characterization of tumor and tissue heterogeneity. By leveraging cell segmentation and morphological feature extraction from mIF imaging, ROICellTrack enables quantitative characterization of cell populations within each ROI, offering a complementary approach to transcriptomic-based deconvolution. Our framework enhances the biological interpretability of GeoMx data by capturing cellular heterogeneity, spatial clustering patterns, and ligand–receptor interactions that define tumor–stroma crosstalk.

To demonstrate the utility of ROICellTrack, we applied it to spatial transcriptomic data from urothelial carcinoma of the bladder (UCB) and upper tract urothelial carcinoma (UTUC), identifying distinct cancer–immune cell mixtures associated with tumor content and immune infiltration. Despite their histological similarities, UTUC and UCB exhibit significant differences in tumor biology, molecular landscapes, and therapeutic responses. While UCB accounts for the majority of urothelial cancers and its tumor microenvironment is well characterized, particularly in the context of intravesical therapies like Bacillus Calmette-Guérin, UTUC is often diagnosed at more advanced stages with a higher propensity for invasion and distinct genomic alterations, including FGFR3 mutations and DNA damage response pathways (Robinson *et al.* 2019, Li *et al.* 2024). However, a comprehensive understanding of the transcriptomic differences between these two entities remains elusive, limiting our ability to develop therapeutic strategies tailored to their distinct microenvironmental features. This study addresses this knowledge gap by leveraging spatial transcriptomic technologies to perform a comparative and integrative analysis of UTUC and UCB, uncovering key differences in their tumor–immune interactions and cellular architectures. By integrating imaging-based cellular segmentation with transcriptomic profiling, our approach identifies distinct spatial patterns of immune infiltration, tumor–stroma organization, and receptor–ligand (RL) interactions unique to each disease and each patient. Our analysis reveals that certain tumor–stroma regions exhibit distinct clustering patterns, with highly mixed tumor–stroma interfaces showing increased immune infiltration and activation of interferon response pathways, whereas more spatially segregated tumor regions are enriched in metabolic stress-related pathways. Furthermore, we find that RL interactions associated with immune signaling are more pronounced in tumor–stroma mixture regions, suggesting potential mechanisms of tumor–immune crosstalk that may shape disease progression and therapeutic response. These findings have therapeutic implications, and future expanded studies with larger cohorts and multimodal spatial omics will be essential to validate and translate them into clinically actionable strategies.

## 2 Materials and methods

### 2.1 ROICellTrack

The analysis toolkit uses ROI images from the GeoMx DSP platform as the input. In the GeoMx DSP Analysis Suite (version 3.0.0.113 used in this analysis), ROI images can be exported by navigating to the left panel in the slide view, selecting “Export Image,” choosing “ROI Report” in the Export options, and then selecting “TIFF” (Tag Image File Format) as the Format. This step will generate square TIFF images with the segmented ROI centered within each image. In our example data, each circular ROI segment is approximately 300 µm in diameter, and the output image is around 600 µm by 600 µm in size. The code provided in the ROICellTrack toolkit then performs three main steps: (i) python code to facilitate automatic image cropping and zooming, removing all content outside of the ROI (including the white margin); (ii) python code for cell segmentation based on a deep neural network, which outputs cell counts, locations, and cellular morphological features; and (iii) R code for calculation of spatial statistics (e.g. cross-K intersection) based on the cell coordinates and cell labels. ROICellTrack is publicly available at https://github.com/wanglab1/ROICellTrack. Additional downstream analysis codes are provided at https://github.com/XiaofeiSong/GeoMx_analysis.

The cell segmentation of ROI images is based on neural network models implemented in Cellpose v2.2.3 (Stringer *et al.* 2021, Pachitariu and Stringer 2022). Cellpose supports both a graphical user interface (GUI) for manual parameter tuning and a command-line mode for automated batch processing. In this analysis, we use the pretrained TissueNet models by setting model_type=“TN3”. Before running segmentation, we recommend using the Cellpose GUI to preview the results and optimize the parameters. The two key parameters include “cell diameter” (set to 23 by default in the pipeline) and “flow_threshold” set to 0.4 by default. For the main segmentation step of cancer cells in our data, we specify channels = [2, 3] to indicate blue-stained nuclei and green-stained cancer cells (PanCK). To help with cell typing after cell segmentation, the visualization of the density of average color intensities (green/red/blue) for each cell and the clustering plots of red and green intensities based on K-means and Gaussian mixture model (GMM) clustering are also performed. In our analysis, we found that GMM better fits the distribution of the red versus green plot and provides better performance in cell classification. We also discovered that the green density has a very clear bimodal distribution, which allows easy dichotomization using an approximate cutoff of 20 in our data. The analysis pipeline will automatically output a snapshot of the original image and cell-annotated segmented images, annotated with total number of cells, cancer cell count, and cancer cell percentage. A pandas.DataFrame object will also be saved, storing the calculated cell-level metrics, including red intensities, green intensities, blue intensities, number of pixels, perimeters, areas, circularity, and X, Y coordinates.

### 2.2 Collection of clinical samples

Six FFPE samples from three patients who underwent concomitant radical cystectomy and nephroureterectomy for concurrent UTUC and UCB were identified at Moffitt Cancer Center. For each FFPE specimen, a pathologist identified and selected 18 ROIs based on the lower tract tumor and upper tract tumor locations and the presence of immune cells. mIF was performed on each FFPE specimen, staining for DNA, PanCK, CD20 (B cells), and CD45 (immunocytes). The GeoMx™ Digital Spatial Profiling platform was used to generate RNA profiles from the ROIs on each FFPE slide.

### 2.3 Pathologist annotations

In this study, two board-certified pathologists independently reviewed the GeoMx Digital Spatial Profiling dataset to ensure independent and accurate ROI annotations. The first pathologist provided primary classifications of ROIs, categorizing them into broad compartments such as tumor (Tu), stroma (St), and mixed tumor–stroma (TuSt) based on histological features and immune infiltration status. The second pathologist conducted a detailed and independent manual review of all ROI mIF images, providing a more granular estimate of tumor cell proportions within each region to better characterize intra-ROI heterogeneity and enable benchmarking with ROICellTrack results. These comprehensive annotations are summarized in Supplementary Table S3.

### 2.4 Spatial clustering

Inspired by Feng *et al.*’s (2023) work, the cross-K area under the curve (AUC) score was used to assess the spatial clustering degree between two types of cells—tumors and nontumors, as defined by ROICellTrack. The cross-K function, implemented in the spatstat R package, was utilized to compare observed patterns with a reference Poisson process. The AUC score was determined by calculating the area between the curve of the cross-K function and the reference pattern.

### 2.5 Other spatial transcriptomics analyses

*Data generation*: Whole transcriptomic profiling and immunostaining were performed using the Nanostring GeoMX^®^-DSP RNA Assay kit (NanoString Technologies, Seattle, WA) on the BOND RX autostainer (Leica Biosystems, Vista, CA). FFPE tissue sections were baked at 60°C for 60 min before being transferred to the BOND RX (Leica Biosystems). The deparaffinization, antigen retrieval, and hybridization were carried out using the fully automated GeoMX DSP FFPE RNA Assay Protocol (NanoString Technologies). Subsequently, slides were washed and stained with morphology markers PanCK, CD45, and Syto13 for 2 h. ROIs were annotated by a pathologist to include tumor cores, stroma regions, and tumor–stroma margins and were placed on 20× fluorescent images scanned by GeoMx^®^ DSP. The photocleaved oligos from the spatially resolved ROIs in the microplate were quantified using standard next-generation sequencing Illumina^®^ workflows. *Data processing*: Downstream bioinformatics analysis was performed using R packages GeoMxTools and NanoStringNCTools. Sequencing quality was assessed for each segment and probe. A gene-level count matrix was generated by averaging corresponding probes per segment. Segments with very few detected genes (<5% of the total detected genes) would be filtered out, and only genes detected in at least 10% of the ROIs were retained. Following these criteria, data from all 56 ROIs and 11 450 genes were retained. The filtered data were then normalized using upper quartile (Q3) normalization. *Deconvolution analysis*: R package SpatialDecon was used to deconvolve the cellular composition of each ROI. To estimate not only stroma and immune cells but also tumor abundance, we utilized a prebuilt reference matrix SafeTME, along with tumor-specific signatures identified from our tumor core sample. The results were visualized by stacked barplot using the R package ComplexHeatmap. *Differential expression test*: The differences in overall transcriptomic landscape across ROIs were examined with uniform manifold approximation and projection (UMAP) dimension reduction and unsupervised clustering. Differential gene expression between groups of interest was tested with a linear mixed-effect model. *RL analysis*: A method was developed on GeoMx data to predict RL pairs by examining the Pearson correlation between ligand–target gene pairs generated by the R package NicheNetb (Browaeys *et al.* 2020, Eyers *et al.* 2024). This method was applied to Mixture and Separative ROIs separately. Specifically, a gene expression matrix was first generated for each type of ROI, and then the pairwise Pearson correlation for any pair of genes was tested. The data were then filtered with a correlation >0.75, and gene pairs documented in NicheNet that are not based on protein–protein interaction (PPI) prediction were kept. Data were visualized using the R package CCPlotR (Ennis *et al.* 2023).

### 2.6 Additional GeoMx datasets

To evaluate the adaptability of ROICellTrack across different tissue types and biomarker staining conditions, we analyzed publicly available GeoMx Spatial Organ Atlas datasets. Two datasets were selected for this analysis: (i) a pancreas dataset, where epithelial cells were stained using PanCK and (2) a lymph node dataset from a breast cancer patient, where CD3-positive immune cells were labeled. These datasets were downloaded from NanoString’s publicly available GeoMx Spatial Organ Atlas (https://nanostring.com/products/geomx-digital-spatial-profiler/spatial-organ-atlas/). For both datasets, we applied ROICellTrack following the same preprocessing steps as in our primary bladder cancer analysis, including image cropping, cell segmentation, and feature extraction.

## 3 Results

**Figure 1.**
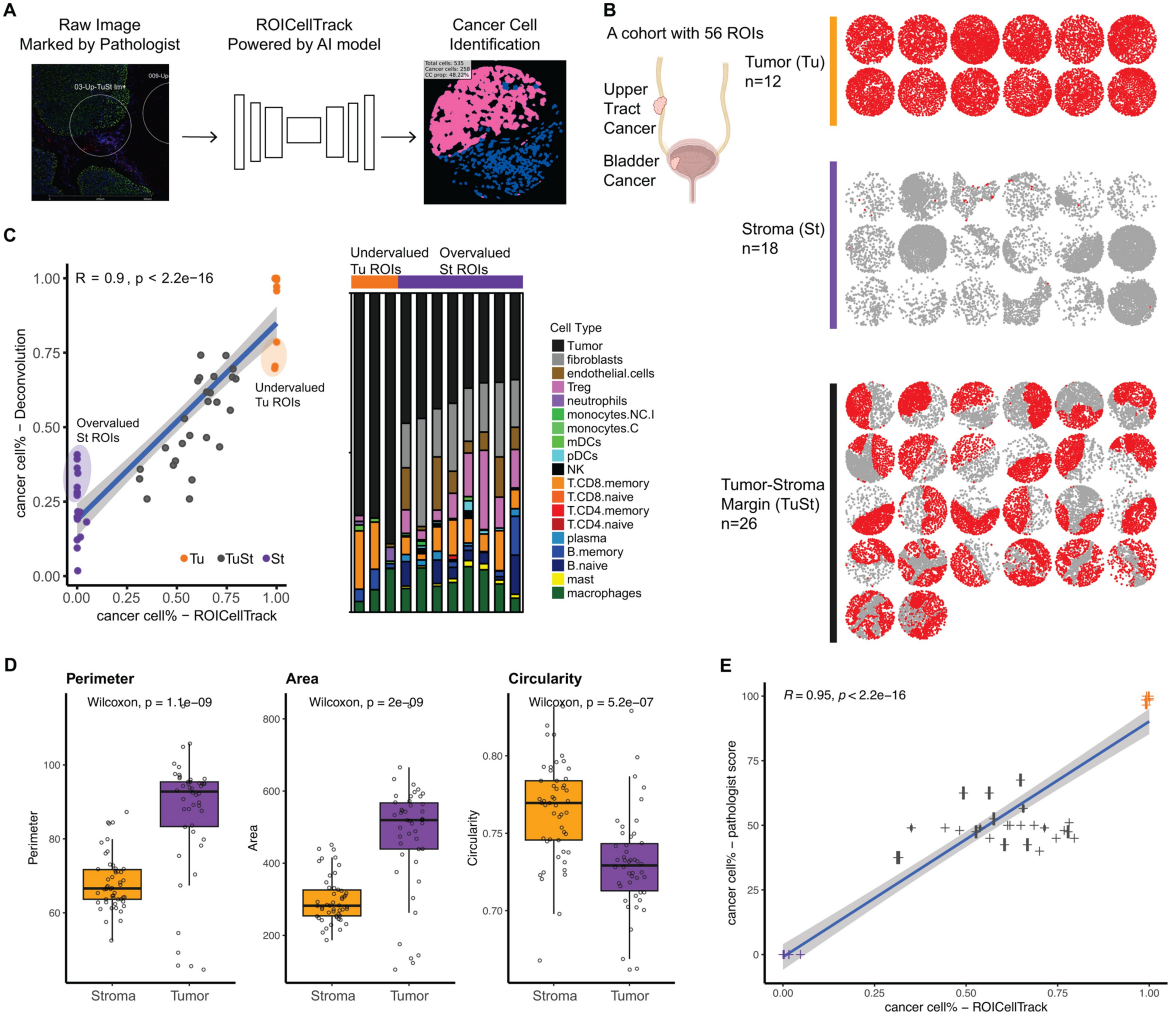
Leveraging spatial transcriptomic profiling with cellular imaging modality. (A) Schematic representation of ROICellTrack, a deep learning framework designed to identify cancer cells and extract image features. (B) Summary of a bladder cancer cohort with 56 regions of interest (ROIs). The ROIs were selected to target three distinct types of regions: tumor cores (Tu), stroma (St), and tumor–stroma margins (TuSt). Each ROI is visualized using point patterns, with each dot representing an individual cell. Cancer cells are distinctly marked for visualization. (C) Comparative performance analysis of ROICellTrack and a gene expression-based cell deconvolution approach. Left panel: correlation analysis between the two deconvolution methods. St ROIs with overestimated tumor purity and Tu ROIs with underestimated purity are circled. Right panel: gene expression-based deconvolution results. The predicted cell-type composition of each sample is presented as a stacked bar plot. (D) Boxplots comparing three morphological features in cells segmented (based on ROICellTrack) from tumor versus stromal regions. Each dot in the plot represents the mean morphological values calculated from the same ROI. (E) Correlation between the ROICellTrack-estimated cancer cell proportions (*x*-axis) and the corresponding values annotated by the second pathologist (*y*-axis). Each dot represents a single ROI, with the horizontal intervals indicating the proportion range provided by the pathologist (detailed pathologist annotations are provided in Supplementary Table S3). Figure partially created with BioRender.com.

By integrating advanced image-based analysis with ROICellTrack, our framework demonstrates the potential to provide novel insights into cancer cell populations, cell-level morphological features, and the complex interplay between tumor cells and their microenvironment (Fig. 1A).

To demonstrate the capabilities and advantages of integrating spatial information into gene expression analysis, we analyzed data generated from bladder cancer patients. This investigation involved spatial RNA profiling of tumor specimens from individuals diagnosed with both UTUC and UCB, utilizing the GeoMx DSP platform (Fig. 1B). In this study, we identified six FFPE samples from concurrent radical cystectomy and nephroureterectomy performed on three patients. We selected and analyzed a total of 56 evaluable ROIs to explore spatial heterogeneity and its association with gene expression patterns in these areas. Each ROI was carefully chosen by the study pathologist to ensure representative coverage of both lower tract and upper tract locations. This selection process also aimed to include a diverse spectrum of the tumor microenvironment: including Tu cores, St, TuSt margins, and regions with high immune cell infiltrates. These ROIs provide useful data essential for benchmarking the estimation of tumor content and cellular composition. mIF was performed on each FFPE specimen with stains for DNA SYTO13, PanCK, CD20, and CD45. The GeoMx DSP platform was used to capture the RNA reads from the ROIs on each FFPE slide. After the initial GeoMx DSP analysis, a second pathologist independently reviewed the ROI images and provided more detailed tumor cell proportion estimates. This effort allowed for a more precise assessment of intratumoral heterogeneity within ROIs and benchmarking with results generated from the proposed pipeline.

To facilitate the analysis of image data from DSP ROIs, we created a toolkit named ROICellTrack. This toolkit streamlines the entire workflow of subcellular image analysis, integrating processes from image preprocessing to cell segmentation and downstream quantitative analysis. As shown in Fig. 1A, the initial step in the ROICellTrack workflow involves the cropping of images to focus on the ROI. While the DSP analytic platform facilitates the batch download of zoomed-in images centered around the ROIs, ROICellTrack enhances this process by further cropping these images to retain only the regions within the ROI that match a predefined shape, typically a circular shape as shown in our study. The main cell identification pipeline was built based on Cellpose, a state-of-the-art cellular segmentation model based on U-Net neural network architecture. Following segmentation, the cells identified within the ROI can be visualized using postsegmentation plots. These plots can be presented through a cell mask view, which aids in analyzing cell size by providing a clear representation of each cell’s shape and area. Alternatively, visualization can be achieved through a cell boundary view, which focuses on the outlines of cells, thereby facilitating the examination of cellular compartments and their spatial relationships.

Following cell segmentation, our next steps involve quantifying cell numbers and analyzing the average color intensities within each cell. As a demonstration of this process, we focused on three-color channels: green representing PanCK (indicative of cancer cells), red for CD45 (marking immune cells), and blue for DNA. The distribution plot of these three-color channels proved highly informative. Notably, the green channel exhibited a bimodal distribution, which serves as a basis for major cell typing, distinguishing between cancerous and noncancerous cells. The red color channel, representing immune cells, does not display a bimodal distribution as visually inspected, primarily due to the inherent blending of red with other colors within the image and impacted by the overall warm color (like reds and yellows) bias of the input image. To further refine our cell typing, we applied clustering methods, specifically K-means and GMM clustering, focusing on the green and red channels. Our findings indicated that GMM clustering yielded more definitive results compared to K-means. Because the green channel’s intensity is effectively clustered into groups, it allows us to build a clustering-free method and establish a simple cutoff at an intensity value of 10 to separate the two cell groups. As shown in the right panel of Fig. 1A, utilizing our cell typing strategy, we were able to estimate that out of a total cell count of 535 within the example regions, 258 were identified as cancer cells. Consequently, the proportion of cancer cells in relation to the total cell population is calculated at 48.22%. This figure offers an absolute measure of the cancer cell burden within the sample, presenting a more accurate assessment compared to relative methods dependent only on total color intensities. ROICellTrack is designed to automatically generate detailed morphological features at the cell level. These features encompass R/G/B intensities, the total number of pixels within each identified cell, as well as the cell’s perimeter, area, and circularity. The output dataset also offers a wealth of information for detailed cell-level downstream analysis. As shown in Fig. 1D, tumor cells generally exhibit a larger perimeter and area, whereas stromal cells tend to be more circular. This observation is expected, as tumor cells often develop irregular shapes due to increased proliferation, genetic alterations, and metabolic reprogramming.

To further validate the accuracy of ROICellTrack, we compared its cell segmentation and positive cell quantification performance against QuPath, an established image analysis platform for pathology applications (Bankhead *et al.* 2017). As shown in Supplementary Fig. S1A, ROICellTrack and QuPath exhibit a strong correlation in both total cell counts per ROI (left panel) and the proportions of positively stained cells (right panel). An example ROI analyzed using QuPath’s “Positive Cell Detection utility” is provided in Supplementary Fig. S1B, where false-positive cells are observed near the ROI margins. Additionally, we evaluated the accuracy of ROICellTrack’s cancer cell proportion estimates by comparing them with the pathologist-annotated values, as shown in Fig. 1E. The strong correlation between these estimates highlights the precision of ROICellTrack in quantifying tumor content, further validating its readiness as an automated tool for subcellular analysis.

We performed differential gene expression analysis to identify molecular distinctions between tumor and stromal ROIs. This was complemented by gene set enrichment analysis (GSEA) to determine the key biological pathways underlying. The top differentially expressed genes comparing these ROIs are listed in Supplementary Table S1. As shown in Supplementary Fig. S2, tumor ROIs are significantly enriched for MYC targets, oxidative phosphorylation, glycolysis, and fatty acid metabolism, suggesting a highly active metabolic state that supports rapid proliferation and biosynthetic demands. In contrast, stromal ROIs show enrichment in TGF-β signaling and epithelial–mesenchymal transition pathways, indicating that the stroma plays an active role in facilitating tumor progression by fostering an invasive tumor phenotype and remodeling the extracellular matrix to support potential metastasis.

Using SpatialDecon, we deconvolved each ROI based on expression signatures representing tumor cells, as well as common stromal and immune cell types within the tumor microenvironment. Our analysis revealed a significant positive correlation between ROICellTrack and the deconvolution method (*R *=* *0.9, *P *<* *2.2e−16), although discrepancies were noted. ROICellTrack accurately identified high tumor purity in Tu cores, moderate levels of tumor cells in TuSt ROIs, and low tumor abundance in St cores. In contrast, deconvolution tended to underestimate tumor purity in tumor regions by misclassifying some cancer cells as immune cells, while overestimating tumor abundance in stromal areas, with discrepancies reaching up to 0.41 (Fig. 1C).

**Figure 2.**
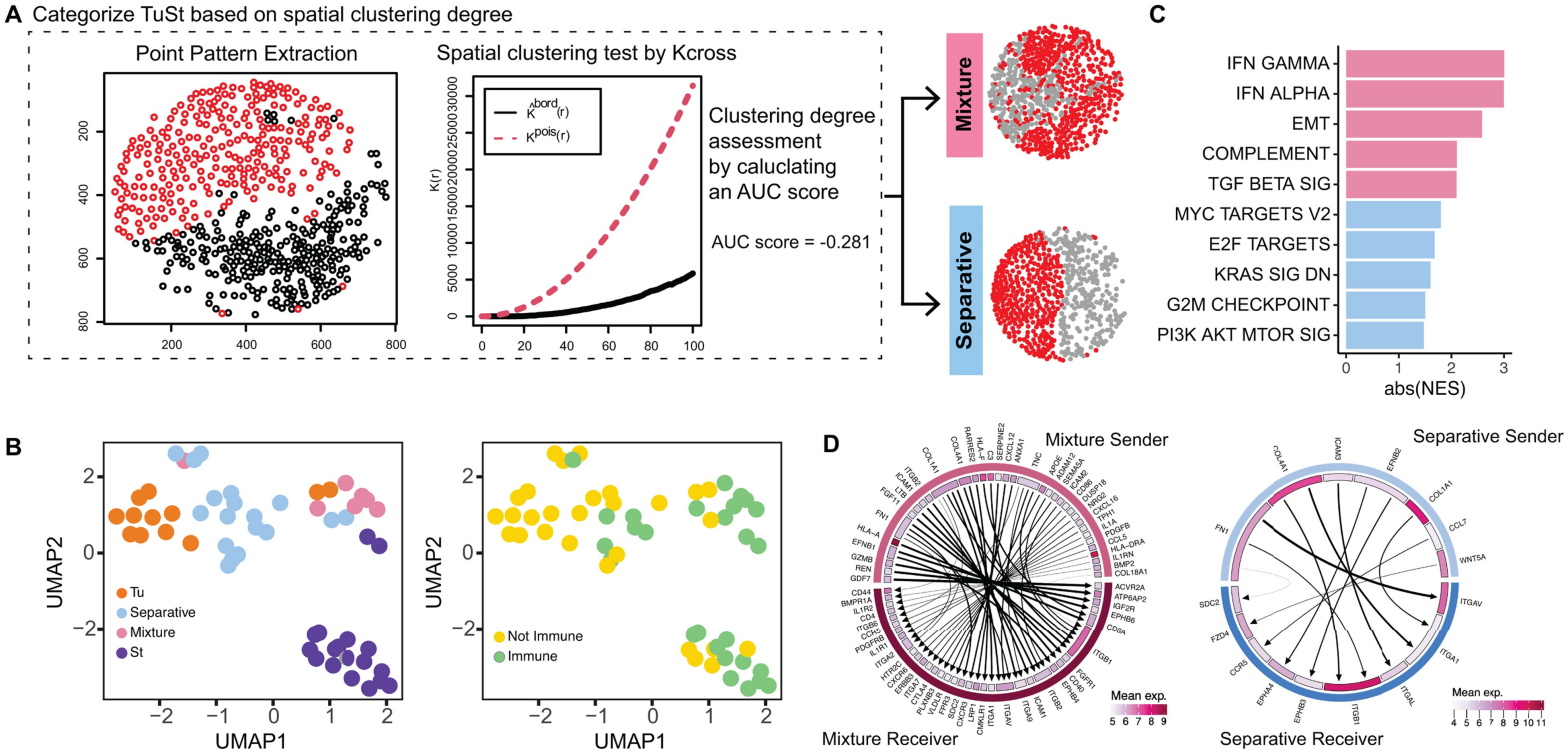
Spatial clustering patterns and associated molecular features in tumor ROIs. (A) The spatial clustering degree was evaluated for an example ROI. The AUC score was determined by calculating the area between the cross-K function curve and the reference Poisson process (dashed line). Margin ROIs were grouped based on the clustering degree into Mixture patterns and Separative patterns. One example ROI for each group is provided. (B) UMAP visualization of ROI clustering by region type (left panel) and immune infiltration (right panel). The immune infiltration status is annotated by a pathologist. (C) Molecular pathways enriched in Mixture ROIs and Separative ROIs. (D) Circos plot showing ligand–receptor pairs within Mixture ROIs (left panel) and Separative ROIs (right panel). Arrows connect gene pairs that are highly correlated, pointing from the ligand to its target gene. The mean normalized expression of each gene is shown in the inner circle.

Beyond assessing tumor purity, we further leverage ROICellTrack to reveal spatial patterns that cannot be inferred from the sequencing modality alone. As illustrated in Fig. 1B, tumor cells may either distinctly separate from stromal cells or blend with them. We hypothesized that various clustering patterns could foster different levels of cell–cell communication, thereby inducing distinct transcriptomic features. To test this hypothesis, we first systematically evaluated the degree of tumor–stroma mixture. By extracting cell distributions as a point pattern, we performed a spatial clustering test using the cross-K function. A larger AUC value indicates a more mixed pattern. We then ranked TuSt ROIs by AUC scores and categorized them into Separative (*n* = 19) and Mixture (*n* = 7). Dimension reduction analysis revealed overall transcriptomic differences among ROIs from different regions (Fig. 2B). Separative ROIs were found to be closer to Tu ROIs on the UMAP plot, whereas Mixture ROIs formed a separate cluster positioned between tumor and stroma. The immune infiltration status was manually examined using H&E and mIF staining by a pathologist. Immune-enriched cores were observed to be positioned separately from immune-depleted cores (Fig. 2A). More interestingly, Mixture ROIs are frequently recognized as immune-enriched, whereas most Separative cores were identified as immune-low/depleted. Motivated by these observations, we performed a differential gene expression analysis comparing Mixture and Separative ROIs. We found that Mixture cores exhibit upregulation of EPSTI1, a gene induced by epithelial–stromal interaction in breast cancer (Nielsen *et al.* 2002), while Separative cores show elevated expression of metabolic stress-associated genes such as B4GALT2 (related to glycolysis) and DHCR24 (involved in cholesterol biosynthesis) (Liu *et al.* 2018). The top differentially expressed genes comparing Mixture and Separative ROIs are listed in Supplementary Table S2. Furthermore, GSEA revealed that the Mixture group exhibits elevated levels of pathways related to interferon-gamma response, interferon-alpha response, and epithelial–mesenchymal transition (Fig. 2C). In contrast, Separative ROIs demonstrate higher activity in pathways associated with cellular proliferation and metabolism, including MYC targets, E2F targets, and G2M checkpoint.

We further adapted a method developed for GeoMx data to predict coregulated RL pairs. Our analysis revealed that Mixture regions exhibit more RL interactions (*n* = 47) compared to Separative regions (*n* = 11, Fig. 2D). RL pairs specific to Mixture regions were enriched in pathways related to cell adhesion molecules, cytokine–receptor interactions, and ECM–receptor interactions. Notably, three MHC-T cell marker pairs (HLA-A-CD8A, HLA-F-CD8A, and HLA-DRA-CD4) were uniquely highly correlated in Mixture regions.

To demonstrate the adaptability of our pipeline across different tissue types and biomarker settings, we applied ROICellTrack to publicly available datasets from the GeoMx Spatial Organ Atlas. As shown in Supplementary Fig. S3, ROICellTrack successfully analyzed pancreatic tissue samples, where epithelial cells stained by PanCK were rendered in yellow. Similarly, in Supplementary Fig. S4, we applied ROICellTrack to lymph node tissue samples from a breast cancer patient, identifying CD3-positive immune cells (rendered in red/pink) within the tumor microenvironment.

## 4 Discussion

While ROICellTrack demonstrates strong performance in cell segmentation, tumor cell proportion estimation, and spatial feature extraction, certain limitations remain. First, cell classification accuracy depends on biomarker staining quality, and performance may vary across datasets with different imaging conditions or marker panels. Future enhancements could incorporate self-supervised learning models trained on diverse spatial transcriptomic datasets to improve cell-type identification in varying experimental settings. Second, while the pipeline currently provides quantitative spatial metrics, it does not yet include direct modeling of spatial dependencies in gene expression patterns. Future iterations could integrate graph-based methods or spatial autoregressive models to better capture cellular interactions and transcriptomic gradients. Finally, further validation across larger and more diverse datasets, including prospective clinical samples, will be essential to establish ROICellTrack’s generalizability and robustness for broader applications in precision oncology and spatial omics research.

In conclusion, our results underscore the importance of integrating *in situ* imaging with spatial transcriptomics for a more accurate and reliable analysis of tumor tissues. Our approach provides critical insights into the tumor microenvironment and cellular interactions, with significant implications for both research and clinical applications in oncology. ROICellTrack could be adapted for newer spatial transcriptomics platforms beyond GeoMx DSP, such as 10× Genomics Visium and Xenium (Janesick *et al.* 2023, Oliveira et al. 2024), which offer single-cell or near-single-cell resolution transcriptomic profiling. The core image analysis and segmentation capabilities of ROICellTrack could serve as a foundation for refining cellular spatial maps in these datasets, improving cell-type annotation and interactions across different platforms. Future work will focus on expanding ROICellTrack’s compatibility with multimodal datasets and developing computational methods that integrate imaging and various omics data. These efforts will include joint models for cell segmentation, segmentation bias detection, spatial clustering, and niche detection.

## Supplementary Material

btaf152_Supplementary_Data

## Data Availability

All codes are available at https://github.com/wanglab1/ROICellTrack. The two public GeoMx datasets are available at https://nanostring.com/products/geomx-digital-spatial-profiler/spatial-organ-atlas/. The bladder cancer GeoMx datasets are available upon request to the authors.
