# Supplementary material for: ROICellTrack: a deep learning framework for integrating cellular imaging modalities in subcellular spatial transcriptomic profiling of tumor tissues
Source: Bioinformatics. 2025 Apr 8;41(5):btaf152. doi: 10.1093/bioinformatics/btaf152 (PMC12085996; doi:10.1093/bioinformatics/btaf152)
Supplement: btaf152_Supplementary_Data [file btaf152_supplementary_data.pdf]

Supplementary materials

ROICellTrack: A deep learning framework for integrating cellular imaging modalities in subcellular spatial transcriptomic profiling of tumor tissues

Xiaofei Song, Xiaoqing Yu, Carlos M Moran-Segura, Hongzhi Xu, Tingyi Li, Joshua T Davis, Aram Vosoughi, G Daniel Grass, Roger Li, and Xuefeng Wang

Supplementary Figures:

- **Supplementary Fig. S1. Comparison of ROICellTrack and QuPath as alternative methods for cell segmentation and positive cell counting.**
- **Supplementary Fig. S2. (A) Volcano plot comparing gene expression profiles of stroma versus tumor cells in the bladder cancer GeoMx dataset. (B) Normalized enrichment scores from GSEA comparing stromal and tumor compartments using the MSigDB Hallmarks gene set**
- **Supplementary Fig. S3. Exemplary results of ROICellTrack applied to a public pancreas dataset downloaded from the GeoMx Spatial Organ Atlas.**
- **Supplementary Fig. S4. Exemplary results of ROICellTrack applied to a lymph node dataset from the Spatial Organ Atlas: Human Lymph Node.**

Supplementary Tables:

- **Supplementary Table S1. Top differentially expressed genes between Tumor (Tu) and Stroma (St) ROIs.**
- **Supplementary Table S2. Top differentially expressed genes comparing ROIs (labeled as TuSt ROIs by the pathologist) that are further identified as "Mixture" versus "Separative".**
- **Supplementary Table S3. Summary of cell metrics from 56 bladder cancer GeoMx ROIs (based on ROICellTrack) and annotations from two board-certified pathologists.**

<u>**Supplementary Figures**</u>

A

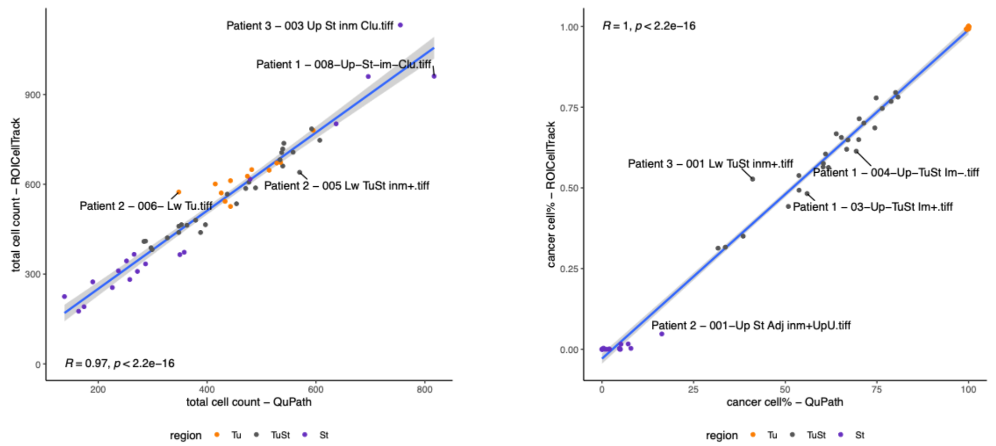

B

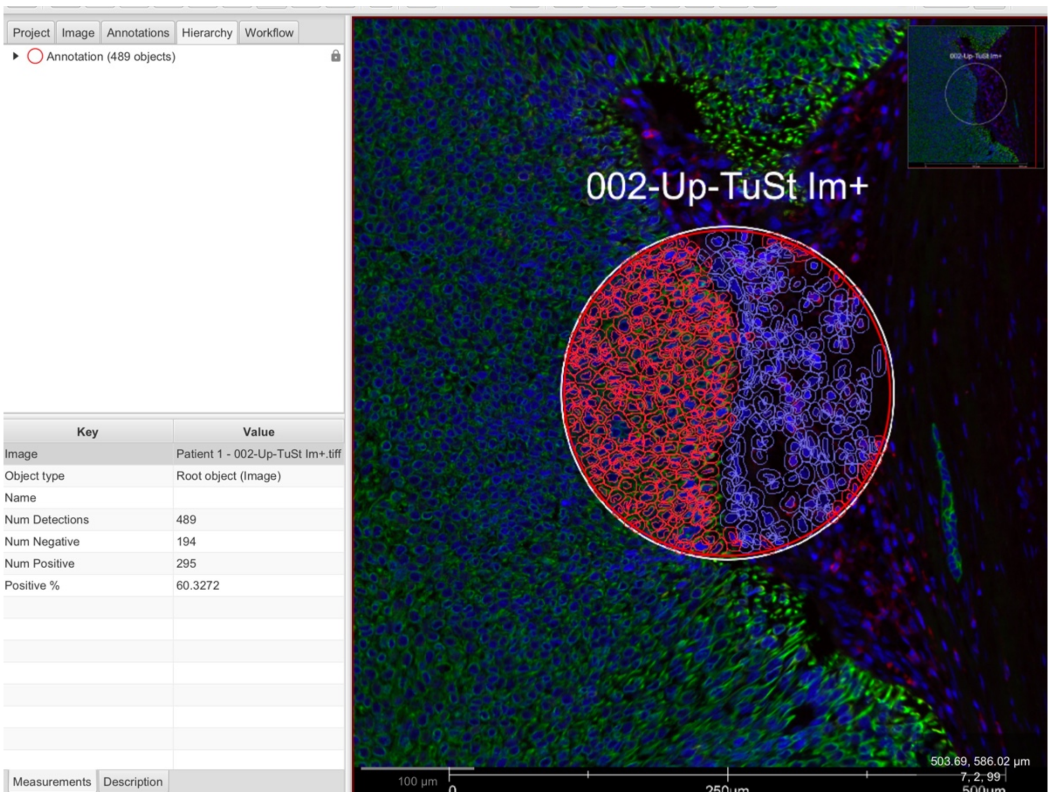

Supplementary Fig. S1. Comparison of ROICellTrack and QuPath as alternative methods for cell segmentation and positive cell counting. (A) The scatterplots demonstrate a high correlation between the two approaches in terms of total cell counts within each ROI (left panel), as well as the number of positively stained cells (right panel). (B) A screenshot showing an example ROI analyzed using QuPath's “Positive Cell Detection” utility. Caveats and details on configuring QuPath for GeoMx ROI analysis are available in the GitHub repository: https://github.com/wanglab1/ROICellTrack.

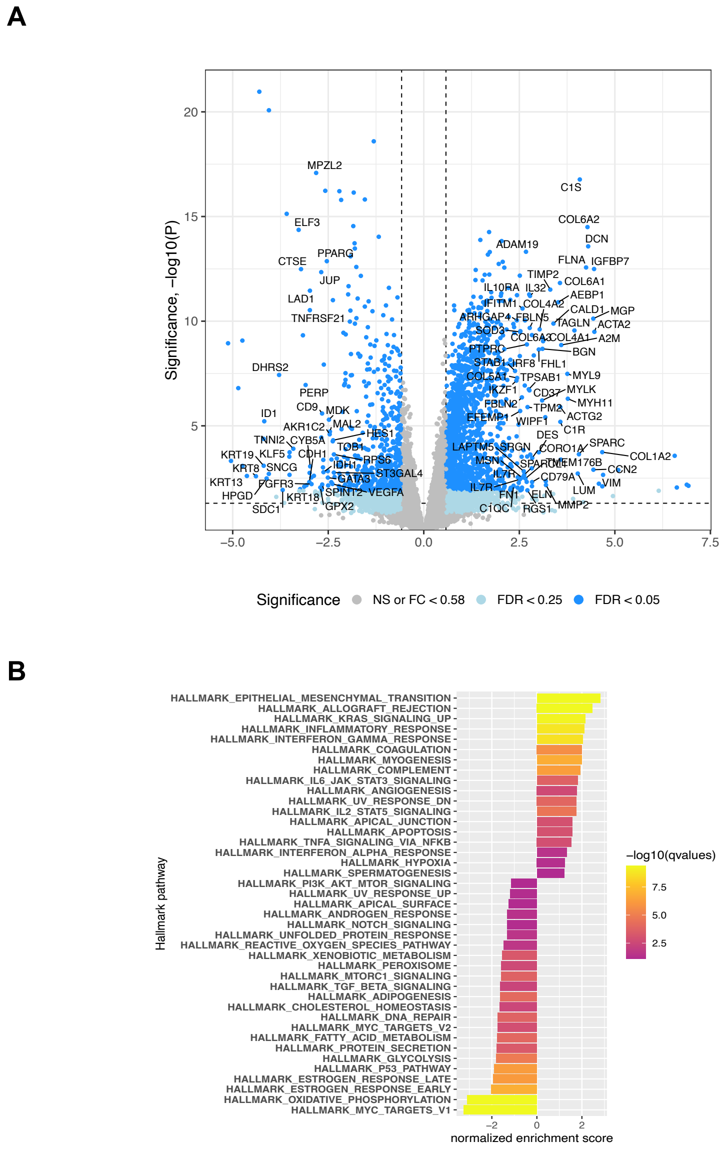

Supplementary Fig. S2. (A) Volcano plot comparing gene expression profiles of stroma versus tumor cells in the bladder cancer GeoMx dataset. The x-axis represents the log2 fold change, where negative values indicate gene upregulation in tumor, and the y-axis represents the −log10 p-value. Highly upregulated or downregulated genes in each compartment are highlighted, with top differentially expressed genes labeled. **(B) Normalized enrichment scores from a gene set enrichment analysis (GSEA) comparing stromal and tumor compartments using the MSigDB Hallmarks gene set.** In tumor ROIs, the enrichment of MYC targets, oxidative phosphorylation, glycolysis, and fatty acid metabolism. In contrast, the enrichment of pathways such as TGF-β signaling and epithelial-mesenchymal transition (EMT) were found in stromal ROIs.

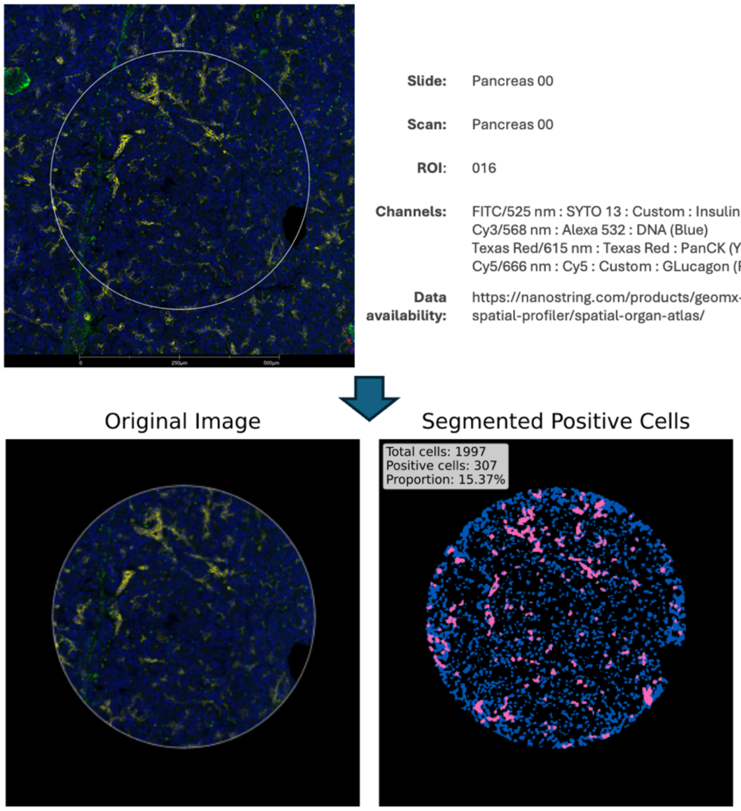

Supplementary Fig. S3. Exemplary results of ROICellTrack applied to a public pancreas dataset downloaded from the GeoMx Spatial Organ Atlas. Epithelial cells are stained by the PanCK biomarker, but rendered as yellow, illustrating how the method can be adapted to different tissue types and biomarker colors.

Slide: hu_lymph_node_003

Scan: 019383T4(1)

ROI: 034(A),035(B),036(C),037(D)

Channels: FITC/525nm : Alexa 488 : SMA (Yellow)
Cy3/568nm : Alexa 532 : DNA (Blue)
Texas Red/615nm : Alexa 594 : CD20 (Green)
Cy5/666nm : Alexa 647 : CD3 (Red)

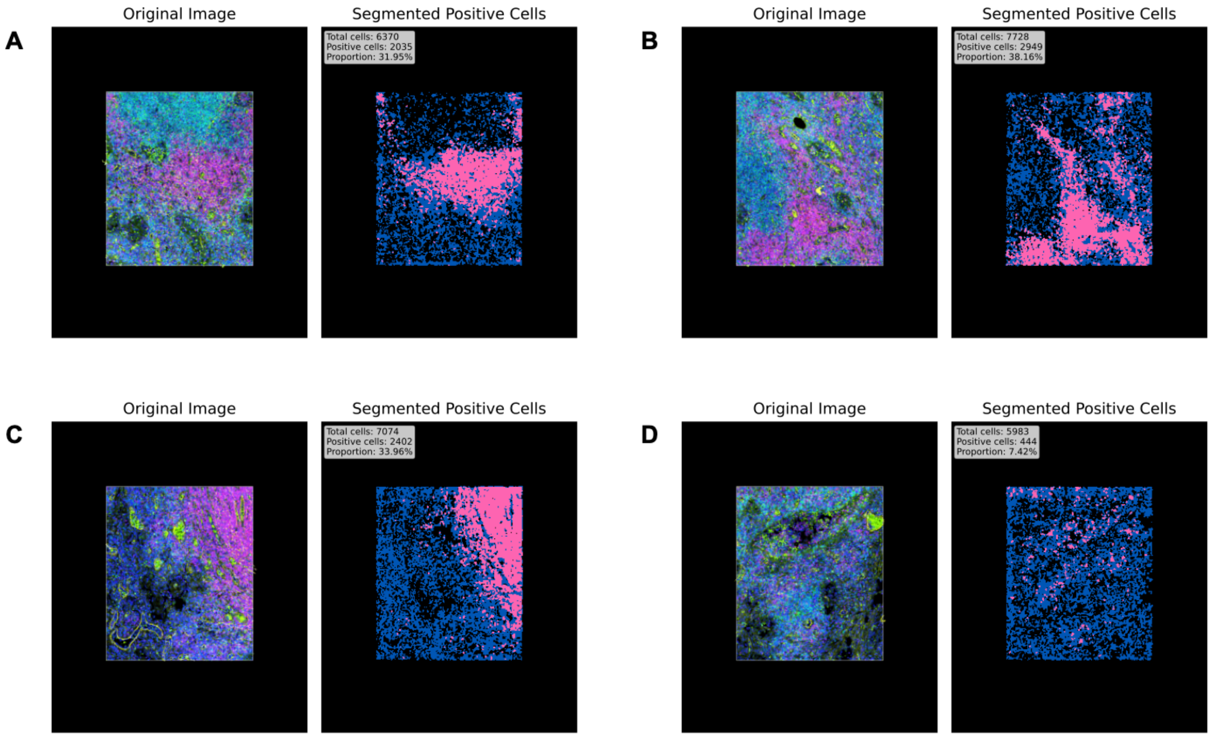

Supplementary Fig. S4. Exemplary results of ROICellTrack applied to a lymph node dataset from the Spatial Organ Atlas: Human Lymph Node (four rectangular ROIs in A-D). The tissue samples are from a patient with breast cancer. Here positive cells are identified by CD3 positive cells (rendered in red/pink).

Supplementary Tables

Supplementary Table S1. Top differentially expressed genes between Tumor (Tu) and Stroma (St) ROIs.

Gene	Up in	log2(FC)	Pr(>|t|)	FDR	hallmark_pathways
VIM	St	4.692	0.002	0.013	EPITHELIAL_MESENCHYMAL_TRANSITION
COL1A2	St	4.670	0.000	0.002	EPITHELIAL_MESENCHYMAL_TRANSITION;UV_RESPONSE_DN
ACTA2	St	4.464	0.000	0.000	EPITHELIAL_MESENCHYMAL_TRANSITION
IGFBP7	St	4.456	0.000	0.000	MYOGENESIS
CCN2	St	4.438	0.001	0.008	EPITHELIAL_MESENCHYMAL_TRANSITION;HYPOXIA
MGP	St	4.435	0.000	0.000	EPITHELIAL_MESENCHYMAL_TRANSITION
DCN	St	4.302	0.000	0.000	APOPTOSIS;EPITHELIAL_MESENCHYMAL_TRANSITION;GLYCOLYSIS;HYPOXIA
COL6A2	St	4.284	0.000	0.000	EPITHELIAL_MESENCHYMAL_TRANSITION;MYOGENESIS
FLNA	St	4.247	0.000	0.000	ALLOGRAFT_REJECTION;EPITHELIAL_MESENCHYMAL_TRANSITION;MITOTIC_SPINDLE
C1S	St	4.083	0.000	0.000	COAGULATION;COMPLEMENT;INTERFERON_ALPHA_RESPONSE;INTERFERON_GAMMA_RESPONSE
SPARC	St	4.060	0.000	0.002	COAGULATION;EPITHELIAL_MESENCHYMAL_TRANSITION;MYOGENESIS
LUM	St	4.029	0.002	0.012	ANGIOGENESIS;APOPTOSIS;EPITHELIAL_MESENCHYMAL_TRANSITION
TAGLN	St	3.942	0.000	0.000	EPITHELIAL_MESENCHYMAL_TRANSITION;MYOGENESIS
MYH11	St	3.769	0.000	0.000	MYOGENESIS
MYL9	St	3.757	0.000	0.000	APICAL_JUNCTION;EPITHELIAL_MESENCHYMAL_TRANSITION
A2M	St	3.597	0.000	0.000	COAGULATION;IL6_JAK_STAT3_SIGNALING
C1R	St	3.574	0.000	0.000	COAGULATION;COMPLEMENT;INTERFERON_GAMMA_RESPONSE
ACTG2	St	3.572	0.000	0.000	APICAL_JUNCTION
COL6A1	St	3.564	0.000	0.000	IL2_STAT5_SIGNALING
AEBP1	St	3.518	0.000	0.000	MYOGENESIS
CALD1	St	3.393	0.000	0.000	EPITHELIAL_MESENCHYMAL_TRANSITION
KRT13	Tu	-4.628	0.002	0.015	ESTROGEN_RESPONSE_EARLY;ESTROGEN_RESPONSE_LATE;KRAS_SIGNALING_DN
KRT8	Tu	-4.392	0.003	0.015	ANDROGEN_RESPONSE;ESTROGEN_RESPONSE_EARLY
KRT19	Tu	-4.189	0.001	0.006	ANDROGEN_RESPONSE;ESTROGEN_RESPONSE_EARLY;ESTROGEN_RESPONSE_LATE
ID1	Tu	-4.174	0.000	0.000	TGF_BETA_SIGNALING;UV_RESPONSE_DN
HPGD	Tu	-4.112	0.003	0.018	ANDROGEN_RESPONSE;FATTY_ACID_METABOLISM
DHRS2	Tu	-3.786	0.000	0.000	ESTROGEN_RESPONSE_EARLY;ESTROGEN_RESPONSE_LATE
SDC1	Tu	-3.692	0.012	0.047	EPITHELIAL_MESENCHYMAL_TRANSITION;GLYCOLYSIS;P53_PATHWAY
SNCG	Tu	-3.513	0.002	0.013	ADIPOGENESIS
KLF5	Tu	-3.512	0.000	0.002	MYOGENESIS
TNNI2	Tu	-3.414	0.000	0.001	MYOGENESIS
ELF3	Tu	-3.274	0.000	0.000	ESTROGEN_RESPONSE_EARLY;GLYCOLYSIS
KRT18	Tu	-3.235	0.011	0.044	APOPTOSIS;ESTROGEN_RESPONSE_EARLY;PROTEIN_SECRETION
CTSE	Tu	-3.212	0.000	0.000	COAGULATION;HEME_METABOLISM
PERP	Tu	-3.092	0.000	0.000	ESTROGEN_RESPONSE_LATE;P53_PATHWAY
CDH1	Tu	-2.994	0.005	0.025	APICAL_JUNCTION;ESTROGEN_RESPONSE_LATE;TGF_BETA_SIGNALING
TNFRSF21	Tu	-2.980	0.000	0.000	ANGIOGENESIS;IL2_STAT5_SIGNALING;IL6_JAK_STAT3_SIGNALING
LAD1	Tu	-2.979	0.000	0.000	ESTROGEN_RESPONSE_EARLY
CYB5A	Tu	-2.973	0.001	0.006	GLYCOLYSIS;OXIDATIVE_PHOSPHORYLATION;XENOBIOTIC_METABOLISM
FGFR3	Tu	-2.937	0.006	0.030	ESTROGEN_RESPONSE_LATE;KRAS_SIGNALING_DN
MPZL2	Tu	-2.817	0.000	0.000	APICAL_JUNCTION;KRAS_SIGNALING_UP

Supplementary Table S2. Top differentially expressed genes comparing ROIs (labeled as TuSt ROIs by the pathologist) that are further identified as “Mixture” versus “Separative.” The “Mixture” and “Separative” classifications are based on the cross K AUC score, as described in the main text.

Gene	Up in	log2(FC)	Pr(>|t|)	FDR	hallmark_pathways
APOC1	Mixture	1.475	0.009	0.508	COAGULATION;COMPLEMENT
RGS5	Mixture	1.260	0.001	0.266	NA
TGFBI	Mixture	1.095	0.008	0.481	RANSITION;GLYCOLYSIS;HYPOXIA
PGGHG	Mixture	1.038	0.009	0.508	NA
EPSTI1	Mixture	1.005	0.000	0.090	AMMA_RESPONSE
FCGR3A	Mixture	0.910	0.019	0.661	NA
ADIRF	Mixture	0.760	0.040	0.817	NA
VAMP5	Mixture	0.715	0.016	0.638	INTERFERON_GAMMA_RESPONSE
SCCPDH	Mixture	0.639	0.049	0.858	NA
PROSER3	Separative	-0.587	0.000	0.024	NA
SLC35E2A	Separative	-0.593	0.028	0.753	NA
NIBAN3	Separative	-0.593	0.002	0.320	NA
SH3D21	Separative	-0.604	0.000	0.235	NA
B3GNT4	Separative	-0.610	0.024	0.720	NA
B4GALT2	Separative	-0.611	0.003	0.348	GLYCOLYSIS
EFS	Separative	-0.613	0.034	0.781	MYOGENESIS
MMP15	Separative	-0.631	0.002	0.311	COAGULATION;COMPLEMENT
PCDH7	Separative	-0.651	0.035	0.784	INFLAMMATORY_RESPONSE
MUC5AC	Separative	-0.725	0.021	0.680	NA
KCTD1	Separative	-0.727	0.000	0.090	NA
TBC1D10A	Separative	-0.729	0.039	0.817	NA
LAMB1	Separative	-0.765	0.003	0.348	NA
ALDH4A1	Separative	-0.787	0.025	0.745	NA
NRG2	Separative	-0.787	0.009	0.497	NA
MS4A1	Separative	-0.795	0.041	0.817	NA
MALL	Separative	-0.830	0.020	0.675	KRAS_SIGNALING_UP
					ON;HYPOXIA;IL6_JAK_STAT3_SIGNALING;P53_PAT
JUN	Separative	-0.850	0.038	0.804	HWAY;TNFA_SIGNALING_VIA_NFKB
DHCR24	Separative	-1.315	0.000	0.085	;FATTY_ACID_METABOLISM;MTORC1_SIGNALING;

Supplementary Table S3. Summary of cell metrics from 56 bladder cancer GeoMx ROIs (based on ROICellTrack) and annotations from two board-certified pathologists.

Patient	Image	ROICellTrack - Cell Detections	ROICellTrack - Tumor count	ROICellTrack (Tumor %)	Pathologist1 (ROI type)	Pathologist1 (immune status)	Pathologist2 (Tumor %)
P1	Patient 1 - 001-Lw-Tu.tiff	528	528	100	Tu	Neg	100
P1	Patient 1 - 001-UP-T-.tiff	595	595	100	Tu	Neg	100
P1	Patient 1 - 002 Lw-Tu.tiff	443	443	100	Tu	Neg	100
P1	Patient 1 - 002-Up-TuSt Im+.tiff	489	295	60.32	TuSt	Pos	45
P1	Patient 1 - 003-Lw-TuSt Im+.tiff	388	259	66.75	TuSt	Pos	50% tumor cells<br>47-49% stromal cells<br>1-3% immune cells
P1	Patient 1 - 03-Up-TuSt Im+.tiff	454	254	55.95	TuSt	Pos	48
P1	Patient 1 - 004-Lw-TuSt Im+ 004.tiff	539	385	71.43	TuSt	Pos	40
P1	Patient 1 - 004-Up-TuSt Im-.tiff	297	206	69.36	TuSt	Neg	50
P1	Patient 1 - 005-Lw-TuSt Im-.tiff	348	259	74.43	TuSt	Neg	50
P1	Patient 1 - 005-Up-TuSt Im1.tiff	397	278	70.03	TuSt	Neg	50
P1	Patient 1 - 006-Lw-TuSt Im-.tiff	379	306	80.74	TuSt	Neg	51
P1	Patient 1 - 006-Up-Tu.tiff	514	514	100	Tu	Neg	100
P1	Patient 1 - 007--Up-TuSt Im-.tiff	299	152	50.84	TuSt	Neg	49
P1	Patient 1 - 007-Lw-St Adj Im+ Im+ 002.tiff	358	17	4.75	St	Pos	0% tumor<br>mostly stromal cells 100%
P1	Patient 1 - 007-Lw-St Adj Im+ Im+.tiff	350	25	7.14	St	Pos	0% tumor cells 5% immune cells
P1	Patient 1 - 008-Up-St-im-Clu.tiff	817	41	5.02	St	Pos	0% tumor 100% immune cells
P1	Patient 1 - 9-Lw-St-im-Clu.tiff	637	31	4.87	NA	NA	0
P1	Patient 1 - 009-Up-St-Adj Im+.tiff	174	9	5.17	St	Pos	0
P1	Patient 1 - 0010-Up-St-Adj Im-.tiff	164	8	4.88	St	Neg	0
Patient	Image	ROICellTrack - Cell Detections	ROICellTrack - Tumor count	ROICellTrack (Tumor %)	Pathologist1 (ROI type)	Pathologist1 (immune status)	Pathologist2 (Tumor %)
P2	Patient 2 - 001-Lw TuSt inm+.tiff	541	182	33.64	TuSt	Pos	35-40% tumor cells<br>60-65% stromal cells
P2	Patient 2 - 001-Up St Adj inm+UpU.tiff	190	31	16.32	St	Pos	100% stromal cells
P2	Patient 2 - 002 Lw TuSt inm+.tiff	539	352	65.31	TuSt	Pos	55-58% tumor<br>rest are stromal
P2	Patient 2 - 002-Up-St Adj Inm +.tiff	266	21	7.89	St	Pos	100% stromal cells
P2	Patient 2 - 003 Lw TuSt inm-.tiff	284	224	78.87	TuSt	Neg	48% tumor<br>rest stromal
P2	Patient 2 - 003-Up TuSt inm+.tiff	534	287	53.75	TuSt	Pos	60-65% tumor<br>rest are stromal
P2	Patient 2 - 004 Lw TuSt inm-.tiff	287	175	60.98	TuSt	Neg	40-45% tumor cells<br>55-60% stromal cells
P2	Patient 2 - 004- Up TuSt inm+.tiff	348	215	61.78	TuSt	Pos	60-65% tumor<br>35-40% stromal
P2	Patient 2 - 005 Lw TuSt inm+.tiff	570	400	70.17	TuSt	Pos	48-50% tumor<br>rest are stromal
P2	Patient 2 - 005-Up Tu.tiff	474	471	99.37	Tu	Neg	95-98% of tumor cells
P2	Patient 2 - 006- Lw Tu.tiff	348	348	100	Tu	Neg	100
P2	Patient 2 - 006-Up Tu 002.tiff	443	441	99.59	Tu	Neg	98-99% tumor cells<br>1-2% of non cellular space
P2	Patient 2 - 007 Lw St Adj inm-.tiff	252	5	1.98	St	Neg	10-12% of stromal cells<br>88-90% of non cellular space
P2	Patient 2 - 007 LwTu.tiff	415	415	100	Tu	Neg	99% tumor cells
P2	Patient 2 - 007 Up TuSt inm+.tiff	558	337	60.39	TuSt	Pos	50-55% of tumor cells<br>45% stromal cells<br>scant empty/non-cellular space
P2	Patient 2 - 008 LwSt inm Clu.tiff	696	14	2.01	St	Pos	85-90% Stromal cell<br>rest are non cellular space
P2	Patient 2 - 008-Up TuSt inm+.tiff	471	316	67.09	TuSt	Pos	65-70% tumor cells<br>25-30% stromal cells<br>5% non cellular space
P2	Patient 2 - 009 Lw St Adj Inm+.tiff	237	1	0.42	St	Pos	25% of stromal cells

Patient	Image	ROICellTrack - Cell Detections	ROICellTrack - Tumor count	ROICellTrack (Tumor %)	Pathologist1 (ROI type)	Pathologist1 (immune status)	Pathologist2 (Tumor %)
P3	Patient 3 - 001 Lw TuSt inm+.tiff	477	196	41.09	TuSt	Pos	45-50% tumor<br>5%immune cells<br>45-50% stroma
P3	Patient 3 - 001- Up TuSt inm+.tiff	592	228	38.51	TuSt	Pos	48-50% tumor<br>5-8% immune cells<br>the rest stroma
P3	Patient 3 - 002 Up St Adj inm+.tiff	272	0	0	St	Pos	85% stroma<br>the rest are either stroma or acellular space.
P3	Patient 3 - 002Lw St Adj inm+.tiff	287	1	0.34	St	Pos	stroma 95%<br>5% immune cell
P3	Patient 3 - 003 Lw St inm Clu.tiff	479	4	0.84	St	Pos	50% stroma<br>5% immune cells (red)<br>the rest are possible stroma
P3	Patient 3 - 003 Up St inm Clu.tiff	755	10	1.32	St	Pos	NA
P3	Patient 3 - 004 Lw TuSt inm-.tiff	437	350	80.09	TuSt	Neg	45% tumor 55% stroma
P3	Patient 3 - 004 Up TuSt inm-.tiff	353	264	74.79	TuSt	Neg	45-50% tumor 50-55% stroma
P3	Patient 3 - 005 Up St Adj inm-.tiff	258	0	0	St	Neg	85-95% stroma<br>2% immune cells<br>the rest - could be stroma or acellular space
P3	Patient 3 - 005Lw TuSt inm-.tiff	327	250	76.45	TuSt	Neg	45% tumor<br>55% stroma
P3	Patient 3 - 006 Up TuSt inm+.tiff	537	170	31.66	TuSt	Pos	35-40% tumor<br>5% immune cells<br>the rest are stroma
P3	Patient 3 - 007 Lw St Adj inm-.tiff	138	0	0	St	Neg	5% immune cells<br>the rest are stroma
P3	Patient 3 - 007 Up TuSt inm-.tiff	363	232	63.91	TuSt	Neg	40-45% tumor<br>54-55% stroma<br>1% immune
P3	Patient 3 - 008 Up St Adj inm-.tiff	226	0	0	St	Neg	99% stroma<br>1% immune cells
P3	Patient 3 - 009 Up Tu.tiff	535	535	100	Tu	Neg	100% tumor
P3	Patient 3 - 009Lox TuSt inm+.tiff	607	326	53.71	TuSt	Pos	48-50% tumor<br>3% immune cells<br>the rest are stoma
P3	Patient 3 - 010 Low Tu.tiff	433	433	100	Tu	Neg	98-99% tumor
P3	Patient 3 - 010 UpTu.tiff	482	482	100	Tu	Neg	100% tumor
P3	Patient 3 - 011 Lw Tu.tiff	426	426	100	Tu	Neg	100% tumor
